# Automated recognition and analysis of body bending behavior in *C. elegans*

**DOI:** 10.1186/s12859-023-05307-y

**Published:** 2023-04-28

**Authors:** Hui Zhang, Weiyang Chen

**Affiliations:** 1 School of Cyber Science and Engineering, Qufu Normal University, Qufu, China; 2 School of Computer Science and Technology, Qilu University of Technology (Shandong Academy of Sciences), Jinan, 250353 China

**Keywords:** *Caenorhabditis elegans*, Body bending, Automatic count, Vitality

## Abstract

**Background:**

Locomotion behaviors of *Caenorhabditis elegans* play an important role in drug activity screening, anti-aging research, and toxicological assessment. Previous studies have provided important insights into drug activity screening, anti-aging, and toxicological research by manually counting the number of body bends. However, manual counting is often low-throughput and takes a lot of time and manpower. And it is easy to cause artificial bias and error in counting results.

**Results:**

In this paper, an algorithm is proposed for automatic counting and analysis of the body bending behavior of nematodes. First of all, the numerical coordinate regression method with convolutional neural network is used to obtain the head and tail coordinates. Next, curvature-based feature point extraction algorithm is used to calculate the feature points of the nematode centerline. Then the maximum distance between the peak point and the straight line between the pharynx and the tail is calculated. The number of body bends is counted according to the change in the maximum distance per frame.

**Conclusion:**

Experiments are performed to prove the effectiveness of the proposed algorithm. The accuracy of head coordinate prediction is 0.993, and the accuracy of tail coordinate prediction is 0.990. The Pearson correlation coefficient between the results of the automatic count and manual count of the number of body bends is 0.998 and the mean absolute error is 1.931. Different strains of nematodes are selected to analyze differences in body bending behavior, demonstrating a relationship between nematode vitality and lifespan. The code is freely available at https://github.com/hthana/Body-Bend-Count.

**Supplementary Information:**

The online version contains supplementary material available at 10.1186/s12859-023-05307-y.

## Background

Nematodes are diverse, with about 40 million species [1]. *Caenorhabditis elegans* (*C. elegans*) is a small worm, one of the few free-living species of linear animals. *Caenorhabditis elegans* is a linear bacteria-eating animal with a body length of 1 mm and transparent body. It is a non-toxic hermaphrodite nematode that lives in soil and feeds on bacteria. Compared with other model organisms, *C. elegans* has the advantages of simple structure, clear genetic background, short life cycle, strong reproductive capacity, multiple sensitive detection indicators, and easy to be cultured in the laboratory [2–4]. Because of these unique advantages, nematodes have become one of the most widely used model organisms in the field of life science research. Nematodes have been widely used in toxicology research, drug screening, aging, and neuroscience [5–10].

*Caenorhabditis elegans* is the first multicellular eukaryote to be sequenced. Its genome has high homology with the human genome and is associated with many human diseases. Therefore, it has unique advantages in drug activity screening and action mechanism research. Its short life cycle is often used in anti-aging research. Anti-aging drugs can be screened and evaluated by observing physiological indicators such as nematode lifespan, head thrash frequency, body bend frequency, pharyngeal pump frequency, and antioxidant damage ability. In addition, since it is very sensitive to exogenous compounds, it has great advantages in toxicological research and is mostly used for toxicity assessment of some metals and organic pollutants [11, 12].

In recent years, nematodes have become excellent animal models for aging, environmental, and toxicological research. Nematodes contain 302 neurons, and their neuronal lineages have been thoroughly described. Locomotive behavior is a rapid indicator to evaluate whether the nervous system of nematodes is damaged [13]. In addition, nematodes are very sensitive to environmental poisons. In previous studies, head thrashing frequency, body bending frequency, and pharyngeal pumping frequency have often been selected to evaluate the locomotion capacity of nematodes [14, 15]. In toxicological studies, nematodes are exposed to toxins, and their locomotion ability is judged by recording the frequency of head thrashing, body bending, and pharyngeal pumping, to further assess the intensity of toxicity. In some anti-aging research, the vitality of nematodes is often assessed by recording these locomotive behaviors. Therefore, these locomotive behavior indicators of nematodes play an important role in drug activity screening and environmental toxicology assessment.

Previous research using artificial measurements of the number of body bends in nematodes have provided important insights into toxicology [14, 16]. However, manual counting is often low-throughput and takes a lot of time and manpower. And it is easy to cause artificial bias and error in counting results. In many investigations, due to the limitation of time and labor force, the number of experimental samples is often reduced, which is easy to cause certain errors in the final experimental results. In addition, during the manual counting process, some subtle changes are often overlooked by the human eye. These problems pose considerable challenges to the collection of experimental data. For example, in some drug screening experiments, the treatment of a potential drug molecule often results in small phenotypic changes [17]. Therefore, a large number of experimental results are required to obtain reproducible results, which is undoubtedly a great challenge for manual counting. There is no doubt that the use of high-throughput, automated, accurate algorithm counting to replace manual counting is the current trend. To meet these needs, some laboratories have developed worm trackers with high accuracy and high-throughput [18–21]. Swierczek et al. propose a method to quantify the nematode body into sine and cosine functions to calculate the number of body bends, which correspond to the phase advance of π [20]. However, this method cannot accurately count some bending behaviors such as Omega bending, which has certain limitations. Cronin et al. quantify the body bending of nematodes by dividing the body into *n* segments and then calculating the bending angle of each segment to generate an angle matrix [19]. This counting method counts every bend in the nematode body, which does not accord with the calculation standard of the number of body bends of nematode described in [22]. Restif et al. proposed a method to calculate the number of body bends by calculating the curvature of each point on the nematode centerline to generate curvature heat maps. This method scores the number of stripes appearing in the heat map/time [18]. However, comparative experiments show that the counting result of this method is higher than that of standard counting according to reference [22].

Hence, a method is proposed for automatically counting and analyzing the body bending behavior. In the first place, the numerical coordinate regression method with convolutional neural network is used to obtain the head and tail coordinates of nematodes. Next, curvature-based feature point extraction algorithm is used to calculate the key points of the nematode centerline. Including the pharynx, inflection points, and peak points. The vertical distance between each peak point and the straight line between the pharynx and the tail is then calculated. The maximum distance is selected as a reference value for the body bend count and marked positive and negative according to the position of the peak point. Next, the number of body bends is counted according to the change in the maximum distance per frame. That is, when the sign of the maximum distance changes and the absolute value reaches the maximum, the count is pushed forward. Nematode amplitude is also quantified by calculating the angle between the peak point and two adjacent inflection points. Finally, experiments are performed to prove the accuracy and effectiveness of the proposed algorithm. Different strains of nematodes are selected to analyze differences in body bending behavior, demonstrating a relationship between nematodes’ vitality and lifespan.

## Methods

**Fig. 1:**
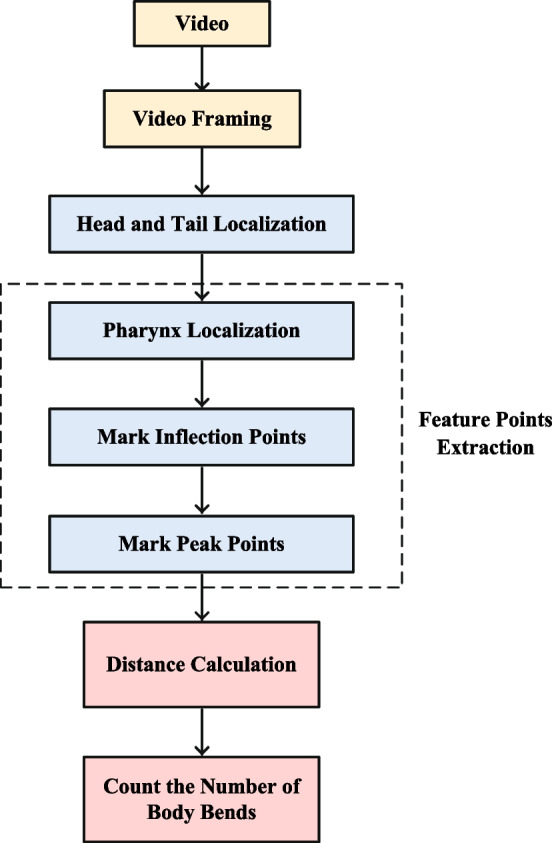
The implementation process of the proposed algorithm

In order to save time and manpower when counting the number of body bends of nematodes. Hence, a method is proposed for automatically counting the number of body bends. In this section, the proposed method is described systematically. First of all, neural network-based head and tail recognition algorithm is applied to locate the head and tail coordinates of nematodes. Next, the feature points of the nematode body centerline are extracted as reference points to calculate the number of body bends, including inflection points and peak points. Finally, a method based on the distance between the peak point of the nematode’s body and the line between the pharynx and the tail is used to calculate the number of body bends. The flow chart of the specific implementation process of the proposed algorithm is shown in Fig. 1.

### Algorithm for head and tail localization

**Fig. 2:**
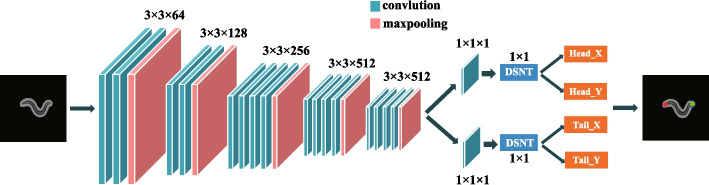
The realization process of head and tail localization algorithm

Firstly, the nematode video is segmented into frames to obtain the original gray image. The objective of the proposed algorithm is to input an image containing nematodes, and finally output the head and tail coordinates of nematodes through a series of processing. The realization process of head and tail localization algorithm is shown in Fig. 2. To be specific, the numerical coordinate regression method with convolutional neural network proposed in [23] is used to obtain the head and tail coordinates of nematodes. Predicting a fixed number of position coordinates corresponding to points of interest marked in the input image is the goal of coordinate regression. Therefore, we first need to manually label the head and tail coordinate information of nematodes and then conduct model training. The proposed method can be spatially generalized and trained end to end using labeled numeric coordinates. First of all, a convolutional network (VGG 19) [24] is used to generate heat maps of a head (*M*_*h*_) and a tail (*M*_*t*_) with a size of 5 × 5. The head and tail of nematodes are spatially represented as heat maps and have higher values. As Fig. 2 shows, in the process of head and tail recognition, all convolution layers are shared except the final convolution layer. This approach not only enables models to share common features but also learns unique features of the head and tail [25]. In addition, it can also save the time consumption of model training.

Since the input of Differential Spatial to Numerical Transform (DSNT) is a single channel normalized heat map [23]. Therefore, the heat maps of the head and tail need to be normalized. That is, all elements of the heat map have to be non-negative and add up to 1. Normalization is accomplished by applying the softmax function on the heat map. The use of the normalized heat map ensures that the predicted coordinates are always within the spatial range of the heat map itself. Finally, the probability of head or tail coordinate positions is given in the normalized heat map. The normalized heat map of the head can be represented as

1 $$M^{\prime}_{h} = softmax\;\left( {M_{h} } \right)$$

The normalized heat map of the tail can be represented as

2 $$M^{\prime}_{t} = softmax\;\left( {M_{t} } \right)$$

DSNT [23] is then used to obtain numerical coordinates of the head and tail from the heat map. Inputs to the DSNT layer are the normalized heat map and the coordinate matrices *X* and *Y.* Each entry of the coordinate matrix *X* and *Y* contains its own horizontal or vertical coordinates, and the scaled image coordinates range between (− 1, 1). A more specific description can be obtained from [23]. The Frobenius inner product operation is used to predict head and tail coordinates. That is, element-wise multiplication of $M^{\prime}_{h}$ and $M^{\prime}_{t}$ with the normalized coordinate matrix, and then the average of the resulting matrix is taken. The head coordinate of nematode is predicted as follows:

3 $$\left( {x_{h} ,y_{h} } \right) = \mu_{h} = \left[ {\left\langle {M^{\prime}_{h} ,X} \right\rangle_{F} ,\left. {\left\langle {M^{\prime}_{h} ,Y} \right\rangle_{F} } \right]} \right.$$

The tail coordinate of nematode is predicted as follows:

4 $$\left( {x_{t} ,y_{t} } \right) = \mu_{t} = \left[ {\left\langle {M^{\prime}_{t} ,X} \right\rangle_{F} ,\left. {\left\langle {M^{\prime}_{t} ,Y} \right\rangle_{F} } \right]} \right.$$

where the prediction *μ* is the mean of the discrete bivariate random vector. Subscript *h* represents the head, and subscript *t* represents the tail. $\left\langle \cdot \right.,\left. \cdot \right\rangle_{F}$ is the Frobenius inner product operation.

Since the output of DSNT layer is normalized coordinate, we chose the mean square error between the predicted coordinate and the ground truth coordinate as the loss [25]. Specifically, the core term of the loss function is formulated by calculating the two-dimensional Euclidean distance between the prediction *μ* and the ground truth *p*. The Euclidean loss function is defined as

5 $$L_{euc} \left( {\mu ,p} \right) = \left\| {p - \mu } \right\|_{2}$$

In order to control the propagation of predictive heat maps, regularization is incorporated into the DSNT loss function, denoted as

6 $$L\left( {M^{\prime},p} \right) = L_{euc} \left( {\mu ,p} \right) + \lambda L_{reg} \left( {M^{\prime}} \right)$$

where *λ* is the regularization coefficient, which is used to set the strength of the regularizer *L*_*reg*_. M′ is a single-channel normalized heat map. Finally, Jensen-Shannon divergence is selected as the optimal regularization term, as described in [25].

To improve efficiency and reduce complexity, the convolutional neural network-based algorithm is used to locate the head and tail coordinates in the first frame. In each subsequent frame, the centerline of the nematode is first obtained by distance-based resample of the dorsal and ventral point sets, and then the coordinates of the two endpoints of the centerline are obtained. The head and tail of the nematode are then located based on the distance between head and head and between tail and tail between two consecutive frames. Among the four head and tail distance components of the nematode coordinates between two consecutive frames, the corresponding head to head and tail to tail distances are the smallest as described in [26].

### Algorithm for feature points extraction

**Fig. 3:**
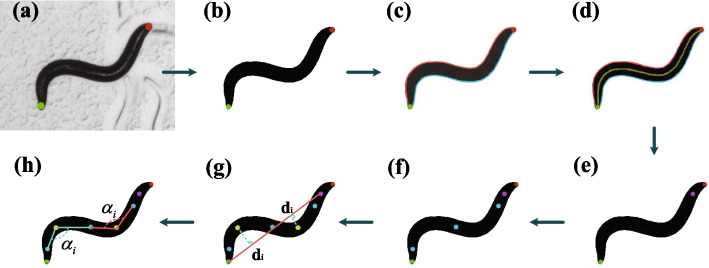
The general process of feature point extraction algorithm. **a** The original grayscale image whose head and tail coordinates have been marked. **b** Clean binary image of nematode. **c** The contour point division of the nematode body. The red side is the dorsal side of the nematode, and the blue side is the ventral side of the nematode. **d** The centerline of nematode. **e** The pharynx of nematode, is marked with a purple dot. **f** The inflection points of the centerline of the nematode's body is marked by blue dots. **g** The peak points of the centerline of the nematode body, are marked by yellow dots. The red line is the line between the pharynx and the tail of the nematode. The vertical distance *d*_*i*_ from the peak point to the line is indicated by a dotted green line. **h** The angle *α*_*i*_ between each peak point and two adjacent inflection points

The head and tail coordinates of nematodes are obtained by the convolutional neural network based head and tail recognition algorithm. As shown in Fig. 3a, the red dot marks the head and the green dot marks the tail. Before feature point extraction, image preprocessing algorithm is used to obtain a clean binary image. To be specific, for images with shadows around nematodes, we first remove the shadows by traversing the pixel values. For an image with a textured pattern as its background, it needs to be first subtracted from the background image to remove unnecessary features. Next, an adaptive local threshold algorithm using a 5 × 5 moving window is used to obtain binary images [26, 27]. The morphological closing operator (binary dilation followed by erosion) [28] is then used to remove small spots in the nematode’s body. The sequential algorithm for component labeling is used to remove small objects in the image [29], resulting in a clean image of nematode, as shown in Fig. 3b.

After binarization, the contour of nematode is extracted. According to the head and tail coordinates of nematode, the contour points of nematode body are divided into ventral and dorsal sides, as shown in Fig. 3c. As Fig. 3c shows, the red side is the dorsal side of the nematode, and the blue side is the ventral side of the nematode. Linear interpolation of a sample size of *n* is used to further resample the two point sets by distance [30]. Finally, dorsal points *D*_*j*_ and ventral points *V*_*i*_ (i = 1, …, *n*) can be obtained. Then, the centerline points *C*_*i*_ of the nematode body are calculated and can be defined as

7 $$C_{i} = \frac{1}{2}\left( {V_{i} + D_{j} } \right),\;i = \left\{ {1, \ldots ,n} \right\}$$

where

8 $$j = \arg \mathop {\min }\limits_{m} \left\{ {\left( {V_{i + k} - V_{i - k} } \right) \cdot \left( {D_{m} - V_{i} } \right)} \right\},\;m = \left\{ {i - r, \ldots ,i + r} \right\}$$

where *k* is the index increment, *r* is the parameter that limits the search area and set *r* = 10. The calculated nematode centerline is shown in Fig. 3d. After defining the centerline points, the nematode length *L* is calculated as

9 $$L = \sum\limits_{i = 1}^{n - 1} {\left| {C_{i + 1} - C_{i} } \right|}$$

In the experiment, we set *n* = 120 to ensure the accurate measurement of nematode length, as described in [30].

The nematode is about 1 mm long [31], and the pharynx of nematode is about 100 μm long [32]. Therefore, we select one-tenth of the length of the nematode centerline as the pharynx point of nematode. As can be seen from Fig. 3e, the purple dot is the pharynx position of nematode. Next, the curvature *κ* of each point on the centerline is calculated and marked positive and negative according to the bump and bump of the body [33]. The inflection point *I*_*a*_ of the centerline is given according to the sign change of curvature *κ*. The inflection point *I*_*a*_ can be defined as

10 $$I_{a} = C_{i}$$

when

11 $$\kappa_{i} \cdot \kappa_{i + 1} < 0$$

The calculated inflection points are shown in Fig. 3f. Next, the maximum value of the absolute value of curvature between two consecutive inflection points is found, called the peak point. As shown in Fig. 3g, the yellow points are the peak points of the nematode body.

In addition, we add an error checking mechanism to check all the calculated feature points. For example, in the calculated characteristic points, the number of inflection points does not correspond to the peak points, and the number of inflection points or peak points is too large. If it is directly used to calculate the number of body bending, it will cause certain deviation. Therefore, we add error checking mechanism to remove the redundant feature points.

### Count the number of body bends

According to the definition in the WormBook [22], every time the part of the nematode just behind the pharynx reaches a maximum bend in the opposite direction from the bend last counted, push the count forward once [22]. First of all, the pharynx and tail of the nematode are connected in a straight line, as shown in Fig. 3g. Next, the vertical distance from each inflection point to the line between the pharynx and the tail is calculated. To facilitate the subsequent calculation, the vertical distance from the peak point to the straight line between the pharynx and the tail is marked with a plus or minus sign. That is, starting from the head of nematode, the peak point in the counterclockwise direction is marked negative. A schematic diagram of the calculated vertical distance is shown in Fig. 3g.

**Fig. 4:**
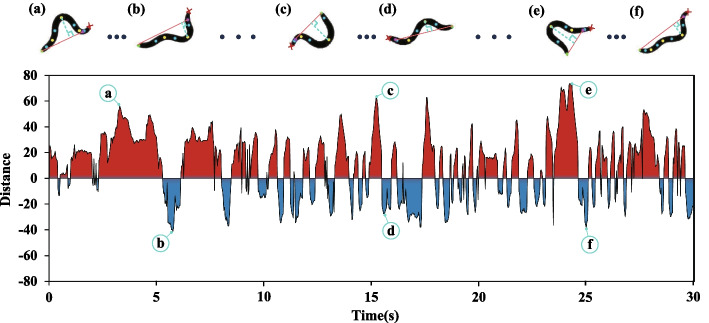
Maximum distance from peak point to the straight line between pharynx and tail. The horizontal axis represents time in seconds. The vertical axis represents the maximum distance. The red part represents the maximum distance in the counterclockwise direction of the head, represented by a plus sign; the blue part represents the maximum distance in the clockwise direction of the head, indicated by a minus sign. The point **a**, **b**, **c**, **d**, **e** and **f** correspond to the locomotion state of the nematode shown in Fig. 4a, b, c, d, e and f respectively

Subsequently, the maximum or minimum value (when the distance is positive, the maximum value is selected; when the distance is negative, the minimum value is selected) of the vertical distance between the peak point and the pharynx and tail in each frame of the image is selected as the reference value for the calculation of the number of body bends. The maximum distance of each frame is shown in Fig. 4. The red part represents the maximum distance in the counterclockwise direction of the head, and the blue part represents the maximum distance in the clockwise direction of the head. As Fig. 4 shows, point a, c, and e corresponding to the locomotion state of the nematode are shown in Fig. 4a, c and e, respectively. At this point, the maximum peak point is in the counterclockwise direction of the nematode head, and the height of the graph is determined by the vertical distance from the peak point to the straight line. The points b, d, and f correspond to the locomotion state of the nematode shown in Fig. 4b, d and f, respectively. Here, the maximum peak point is in the clockwise direction of the nematode head.

The count is pushed forward when the back part of the nematode’s pharynx reaches its maximum bend in the opposite direction from the previous count. For example, in Fig. 4, it can be seen that in a certain period, the nematode body reaches its maximum bend at point a, and then in the opposite direction at point b, the number of body bends of nematode is increased by one. At the same time, the count can continue to advance when the nematode reaches its maximum bend in the opposite direction to point b. In addition, to reduce the error in the counting process, we will ensure that at least three frames near the maximum bending point are in the same direction as the current peak point.

## Results

In the previous section, the implementation process of automatic counting method for nematode body bends is introduced in detail. In this section, experimental results are presented to prove the robustness of the proposed algorithm. In the first place, the accuracy of the head and tail localization algorithm based on convolutional neural network (VGG 19) on the test dataset is verified to prove the superiority of the proposed algorithm. Then, the results of manual counting and automatic counting are compared to verify the accuracy of the proposed counting algorithm. Finally, nematodes of different strains and lifespans are selected to count the number of body bends. The relationship between vitality and longevity of nematodes is explored by comparing and analyzing the experimental results.

### *C. elegans* datasets

In this paper, nematodes in different states are selected from two databases for experiments. First of all, wild type N2(Schafer Lab N2), *egl-30*(*ep271*), *odr-3*(*n2150*), *daf-7*(*m62*), *daf-5*(*e1386*), *daf-3*(*e1376*) and *jnk-1*(*gk7*) of *C. elegans* are selected from the *C. elegans* behavioral phenotypes database [34]. A total of 305 *C. elegans* strains are included in the database, which consists of 9203 short videos. To be more specific, the collection of nematode videos in the database has been maintained under strictly controlled conditions. Camera magnification is set between 3.5 and 4.5 microns/pixel (at 640 × 480 resolution, the corresponding FOV is about 2.5 × 2 mm) [34]. The nematodes photographed are young-adult hermaphrodites that spontaneously behaving on food. At least 20 nematodes are photographed for at least 15 min for each strain. Each video in the database has a frame rate of 20–30 frames per second [34]. Another dataset is for nematodes in the ‘escape response’ condition. Specific filming conditions and cultivation methods are described in [35]. To put it simply, nematodes were recorded in temperature-controlled chambers (22.5 ± 1 °C). Applying a 100 ms, 75 mA infrared laser pulse from a diode laser (*λ* = 1440 nm) to the worm's head increases the temperature within the FWHM-radius of 220 m by about 0.5 °C [35]. Each video has a frame rate of 20 frames per second.

### Verification of head and tail location algorithm

In order to evaluate the effectiveness and accuracy of the head and tail localization algorithm, 1400 grayscale images of nematodes are selected from *C. elegans* behavioral phenotypes database [34]. These images include seven nematodes of different strains in different states. First, we manually tagged the head and tail of the nematode. In the experiment, 70% (980) of the images are used as the training dataset and 30% (420) as the validation dataset. In addition, the learning rate is set to 5e-4, and Adam is selected as the optimizer [25]. The model is trained with 350 epochs and a batch size of 64.

**Fig. 5:**
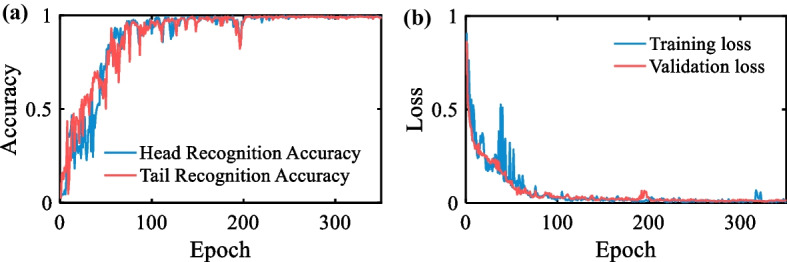
**a** Head and tail recognition accuracy on validation dataset. **b** Train and validation loss comparison

In order to better evaluate the performance of the head and tail localization algorithm, the probability of correct key point accuracy [25] is selected as the judgment standard of the prediction accuracy of the head and tail coordinates. The probability of correct key point accuracy is defined as the percentage of predicted coordinates located within *n* pixels of the ground truth label. In this paper, we set *n* = 8. In the previous calculations, the width of the nematode head and tail is about 5–7 pixels. This value is smaller than the body width of the nematode, so the predicted coordinates do not affect subsequent calculations. In addition, the mean square error (MSE) between the predicted coordinates and the ground truth coordinates is selected as the loss. The accuracy of the algorithm is evaluated on the validation dataset. The accuracy curve of head and tail coordinate prediction is shown in Fig. 5a. The training and validation loss curves for every epoch are shown in Fig. 5b. The accuracy of head coordinate prediction is 0.993, and the accuracy of tail coordinate prediction is 0.991. Experimental results show that the proposed head and tail localization algorithm is robust.

### The proposed algorithm verification by manual counting

**Fig. 6:**
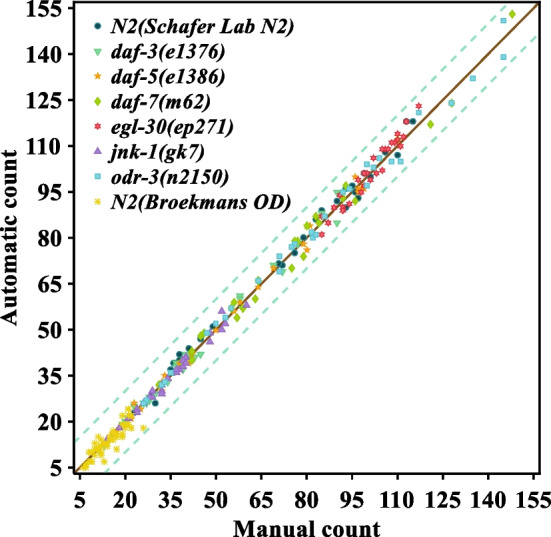
The number of body bends in one minute is counted manually and automatically. The horizontal axis is the result of manual counting and the vertical axis is the result of automatic counting. Points with different colors and shapes respectively represent the results of manual counting and automatic counting of different strains. The solid brown line is a straight line through the origin. The two green dotted lines are error lines with slope 1 and intercept 10

To assess the accuracy of the proposed algorithm, trained human observers are selected to manual count the number of body bends. The results of manual counting and automatic counting are compared. In the first place, 259 one-minute video clips are selected from two nematode databases [34, 35]. Among them, all of the videos are selected for the experiment in the ‘escape response’ database [35]. There are ninety-eight 30 s of videos in this database, which are combined into forty-nine 1 min nematode videos for convenience of counting. In addition, N2 strains in this database are referred to as ‘N2(Broekmans OD)’ for better differentiation. Next, wild type N2(Schafer Lab N2), *egl-30*(*ep271*), *odr-3*(*n2150*), *daf-7*(*m62*), *daf-5*(*e1386*), *daf-3*(*e1376*) and *jnk-1*(*gk7*) of *C. elegans* are selected from the *C. elegans* behavioral phenotypes database [34]. Due to a large number of videos in the database, 30 one-minute video clips are randomly selected for each type of strain to count. Next, all videos are counted manually and automatically. The number of body bends in one minute is counted manually and automatically, as shown in Fig. 6. Specific data information can be found in the Additional file 1. As Fig. 6 shows, points with different colors and shapes respectively represent the results of manual counting and automatic counting of different strains. Eventually, the results of all manual and automatic counting converge around a line that passes through the origin. The Pearson correlation coefficient between the results of the automatic count and manual count of the number of body bends is 0.998 and the mean absolute error is 1.931. Experimental results show that the proposed algorithm has high accuracy and robustness.

### The differences in body bends of different nematode strains

**Fig. 7:**
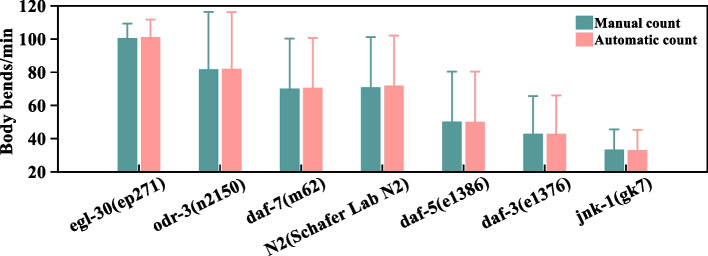
Mean value and standard deviation of the number of body bends per minute for different strains of nematodes

To evaluate differences in body bending between strains, seven groups of nematodes of different strains are selected. Including wild type N2(Schafer Lab N2) of *C. elegans*, *egl-30*(*ep271*), *odr-3*(*n2150*) and *daf-7*(*m62*) of *C. elegans*, which have a longer lifespan; *daf-5*(*e1386*), *daf-3*(*e1376*) and *jnk-1*(*gk7*) of *C. elegans*, which have a shorter lifespan [36–39]. Thirty one-minute videos are randomly selected for each strain to be manual count and automatic count the number of body bends. The mean value and standard deviation of manual count and automatic count for each strain are shown in Fig. 7. More detailed results are shown in Table 1. It can be seen from Fig. 7 that the average number of body bends per minute of N2(Schafer Lab N2) of *C. elegans* is about 71 times by manual count and 72 times by automatic count. The average number of body bends per minute for *egl-30*(*ep271*) of *C. elegans* is about 100 times by manual count and 101 times by automatic count. The *odr-3*(*n2150*) of *C. elegans* averaged about 82 times body bends per minute for both manual and automatic count. The *daf-7*(*m62*) of *C. elegans* averaged about 70 times body bends per minute for both manual and automatic count. The *daf-5*(*e1386*) of *C. elegans* averaged about 50 times body bends per minute for both manual and automatic count. The *daf-3*(*e1376*) of *C. elegans* averaged about 43 times body bends per minute for both manual and automatic count. The *jnk-1*(*gk7*) of *C. elegans* averaged about 33 times body bends per minute for both manual and automatic count.

**Table 1:** Mean and standard deviation of the number of body bends

	Mean (manual count)	Mean (automatic count)	Standard deviation (manual count)	Standard deviation (automatic count)
*egl-30*(*ep271*)	100	101	8.98	10.86
*odr-3*(*n2150*)	82	82	34.81	34.28
*daf-7*(*m62*)	70	70	30.28	30.33
N2(Schafer Lab N2)	71	72	30.38	30.37
*daf-5*(*e1386*)	50	50	30.45	30.36
*daf-3*(*e1376*)	43	43	22.97	23.33
*jnk-1*(*gk7*)	33	33	12.45	12.39

From the experimental results, it can be seen that the *egl-30*(*ep271*), *odr-3*(*n2150*) and *daf-7*(*m62*) of *C. elegans* which have a longer lifespan have higher the number of body bends than the *daf-5*(*e1386*), *daf-3*(*e1376*) and *jnk-1*(*gk7*) of *C. elegans*, which have a shorter lifespan. In previous studies, the number of body bends has often been selected to evaluate locomotion capacity of nematodes [14, 15]. The vitality of nematodes is related to their locomotion rate, and nematodes showed an age-related decline in vitality, which is characterized by reduced physical movement [40–42]. These experimental videos are selected from the same database with the same cultivation and recording conditions. Counting results show that nematodes with longer lifespans have stronger locomotion than nematodes with shorter lifespans under the same conditions. Therefore, it can be inferred that the nematodes with longer lifespans show higher vitality than nematodes with shorter lifespans under the same conditions. In addition, as shown in Fig. 7, it can be seen that each strain has a high standard deviation. This shows that the number of body bends of nematodes varies greatly in different periods.

**Fig. 8:**
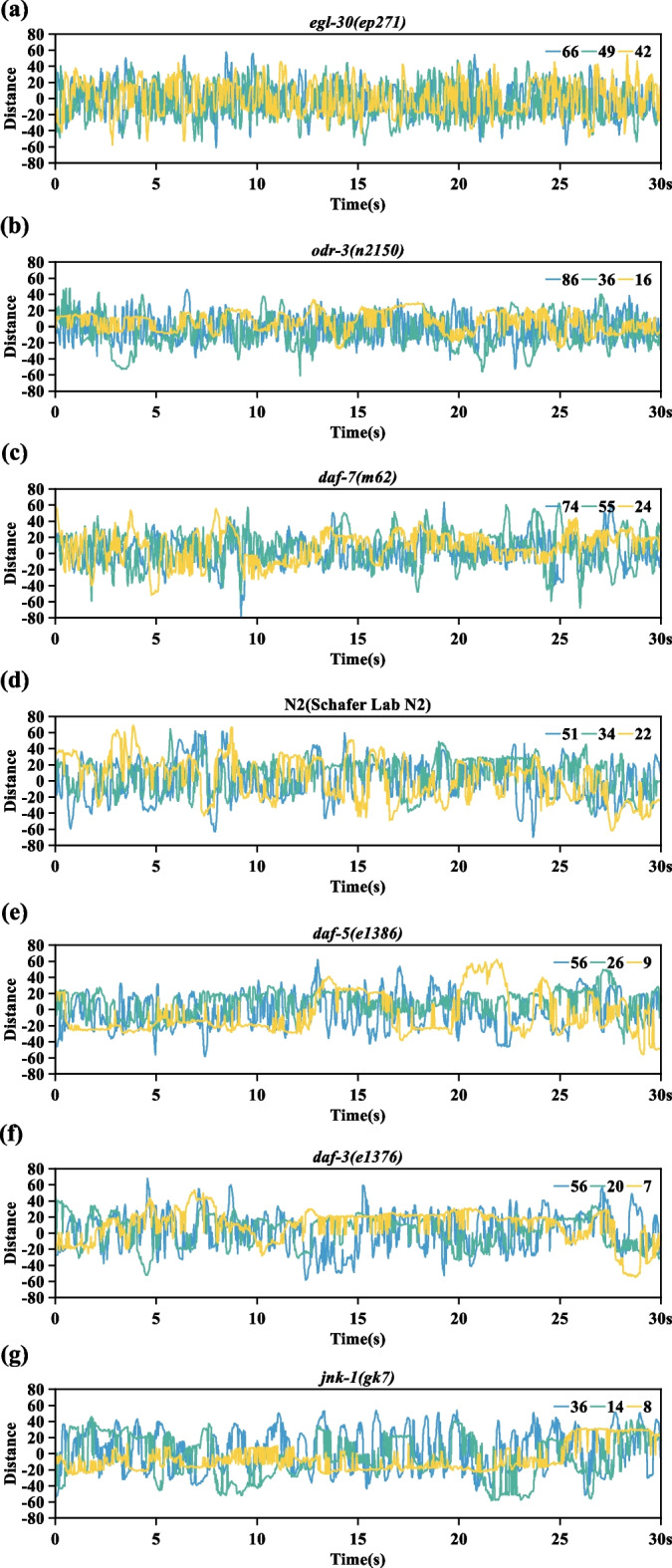
The maximum distance curve from the peak point to the straight line between the pharynx and the tail within 30 s. **a** Time-Distance curve of *egl-30*(*ep271*) of *C. elegans*. The blue, green, and yellow curves indicate 66, 49, and 42 body bends in 30 s. **b** Time-Distance curve of *odr-3*(*n2150*) of *C. elegans*. The blue, green, and yellow curves indicate 86, 36, and 16 body bends in 30 s. **c** Time-Distance curve of *daf-7*(*m62*) of *C. elegans*. The blue, green, and yellow curves indicate 74, 55, and 24 body bends in 30 s. **d** Time-Distance curve of N2(Schafer Lab N2) of *C. elegans*. The blue, green, and yellow curves indicate 51, 34, and 22 body bends in 30 s. **e** Time-Distance curve of *daf-5*(*e1386*) of *C. elegans*. The blue, green, and yellow curves indicate 56, 26, and 9 body bends in 30 s. **f** Time-Distance curve of *daf-3*(*e1376*) of *C. elegans*. The blue, green, and yellow curves indicate 56, 20, and 7 body bends in 30 s. **g** Time-Distance curve of *jnk-1*(*gk7*) of *C. elegans*. The blue, green, and yellow curves indicate 36, 14, and 8 body bends in 30 s

In addition, in order to better illustrate the differences in body bending among different strains of nematodes, the time-distance curve is drawn as shown in Fig. 8. Distance is the maximum vertical distance between the peak point and the straight line between the pharynx and the tail in each frame. If the maximum distance is in the counterclockwise direction of the nematode head, it is marked with a positive sign, and vice versa is marked with a negative sign. The maximum distance here is similar to the maximum amplitude of the worm’s body. It can be seen from Fig. 4a–f that the greater the maximum vertical distance between the peak point and the straight line between the pharynx and the tail, the deeper the body bend of the nematode. Three sets of data are randomly selected from each strain to draw a time-distance curve. The three groups of data are the number of body bends at a high stage, the number of body bends at a medium stage and the number of body bends at a low stage in each strain respectively. As can be seen from Fig. 8, the curve fluctuation of the first three strains (Fig. 8a, b and c) is more intensive, indicating that their body bending frequency is higher than N2(Schafer Lab N2) of *C. elegans* (Fig. 8d). The curves of the last three strains (Fig. 8e, f and g) are relatively sparse, indicating that their body bending frequency is lower than that of N2(Schafer Lab N2) of *C. elegans* (Fig. 8d). Therefore, it can be inferred that the nematodes with longer lifespans show higher vitality than nematodes with shorter lifespans under the same conditions. Nematodes showed an age-related decline in vitality. Furthermore, it can be seen from Fig. 8 that the fluctuation of the curve is variable [40–42]. This indicates that the body bending amplitude of different strains of nematodes is variable at different periods.

## Discussion

In order to verify the robustness of the proposed algorithm, the results compared with related methods are discussed in this section.

### Comparison with other head and tail recognition algorithms

Most previous studies have used the following two methods to recognize the heads and tails of nematodes. The first method is head and tail recognition based on curvature, that is, the head is rounder than the tail [43, 44]; the second method is head and tail recognition based on grayscale, that is, the tail area is darker than the head [26]. Curvature-based head and tail recognition methods usually require preprocessing of nematode images. Edge detection is then performed to find the nematode boundary. Three points are taken on the boundary of binary image, and the least angle is found to be the tail of nematode [43]. However, under common imaging conditions, the tail of the nematode usually does not look sharp enough to cause misidentification. The head and tail recognition method based on gray level needs to calculate the median brightness of the two end parts, and the end region with a higher average brightness value is marked as the head [26]. This method is highly dependent on light conditions in the photographing process of nematode datasets. Under common imaging conditions, the head is often darker than the tail. In addition, some studies usually combine these judgment criteria in header and tail recognition [26, 43], and carry out some artificial error checking mechanism, which requires manual setting of parameters [21].

In order to better analyze the accuracy of the above two methods, we conducted experiments on experimental datasets. We used both the curvature-based and grayscale-based recognition method to recognize the head of the first frame. When these two methods conflict, manual recognition is needed. The head and tail recognition algorithm is tested on the first frame of 210 1 min videos. The experimental results are shown in Table 2. The rate of conflict between curvature-based and grayscale-based head recognition methods is 8.1%. Manual checking is used to ensure that the head recognition in the first frame is correct when conflicts occur. When there is no conflict, the error rate of head recognition is 2.4%.

**Table 2:** Recognition of the head for various strains

Worm type	Number of videos	Number of conflicts^a^	Curvature-based wrong^b^	Grayscale-based wrong^c^	Recognition wrong^d^
*egl-30*(*ep271*)	30	3	2	1	1
*odr-3*(*n2150*)	30	2	1	1	1
*daf-7*(*m62*)	30	2	1	1	1
N2(Schafer Lab N2)	30	0	0	0	0
*daf-5*(*e1386*)	30	3	2	1	2
*daf-3*(*e1376*)	30	4	3	1	0
*jnk-1*(*gk7*)	30	3	1	2	0
Total	210	17	10	7	5

^a^The number of conflict the between curvature-based and grayscale-based head recognition methods

^b^The error that occurred in curvature-based method when conflict occurs

^c^The error that occurred in grayscale-based method when conflict occurs

^d^The error that occurred in recognition of the head when no conflicts occur

In this paper, a head and tail localization algorithm based on convolutional neural network (VGG19) is used [25]. Compared with other related methods [21, 26, 43, 44], the algorithm has the following advantages. First of all, nematode images do not require image preprocessing and retain the characteristics of the original gray image, which improves work efficiency. Secondly, the method does not need to use multiple judgment criteria and error-checking mechanisms at the same time, which reduces the complexity of the automatic. Finally, the algorithm has higher accuracy in predicting the head and tail coordinates. In our experiments, different neural networks are used to predict head and tail coordinates. The accuracy of prediction using different networks is shown in Table 3. It can be seen that compared with the other two networks, the accuracy of using VGG19 is higher. The accuracy of nematode head coordinate prediction is 0.993, and the accuracy of nematode tail coordinate prediction is 0.991.

**Table 3:** The accuracy of coordinate prediction in different neural networks

	Head (percentage accuracy)	Tail (percentage accuracy)
ResNet18	0.950	0.921
VGG16	0.971	0.979
VGG19	0.993	0.991

### Comparison with other automatic counting algorithms

In recent years, several powerful worm trackers have been developed, and these automatics can quantify the bending behavior of nematodes. However, they also have some problems. For example, a real-time computer vision system Multi-Worm Tracker (MWT) is described in [20]. The MWT method calculates the number of body bends by quantifying the nematode body as a sine and cosine function, with a body bend corresponding to an advance of π in phase [20]. This method cannot accurately count some bending behaviors such as Omega bending, which has certain limitations. Some methods quantify the body bending of nematodes by dividing the nematode body into *n* segments and then calculating the bending angle of each segment to generate an angle matrix [19, 34]. The bending frequency is defined as the oscillation frequency between adjacent segments as described in [19]. In the method described in [34], the calculated bending angle for each segment is marked as positive or negative according to the dorsal–ventral direction of the nematode. Then the angle matrix is normalized, and finally the angle is checked from beginning to end. Each time the angle changes sign or reaches 0 degrees, the bent end is found and the count is pushed forward. Even ignoring the small curves at the tips of the head and tail. This counting method counts every bend in the nematode body, which does not accord with the calculation standard of the number of body bends of nematode described in [22].

**Fig. 9:**
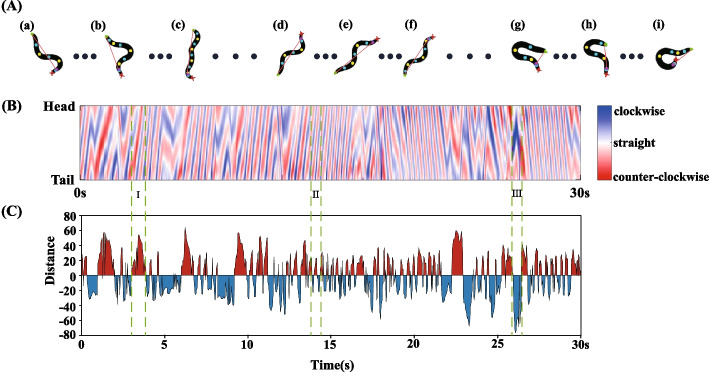
Comparison between the proposed method and CeleST method. **A** Locomotion state of nematodes in different periods. (a)–(c) represents the locomotion state of nematode in the region I; (d)–(f) represents the locomotion state of nematode in region II; (g)–(i) represents the locomotion state of nematode in region III. **B** Curvature heat map of CeleST method counting. The vertical axis represents the curvature of each point of the nematode body, with the curvature of the head at the top and the curvature of the tail at the bottom. **C** Maximum distance from peak point of nematode to straight line between pharynx and tail. The red part represents the maximum distance in the counterclockwise direction of the head, represented by a plus sign; the blue part represents the maximum distance in the clockwise direction of the head, indicated by a minus sign

Most methods of calculating the number of body bends are by calculating the curvature at each point of the nematode's centerline [18, 45, 46]. Take the CeleST method described in reference [18] for example. Specifically, the curvature of the centerline is calculated at multiple locations, followed by applying a one-dimensional Fast Fourier Transform to the curvature at multiple body locations. Subsequently, the number of body bends is calculated from time coordinates in the short-time Fourier analysis of the curvature heat map. This method scores the number of stripes appearing in the heat map/time [18]. The curvature heat map of CeleST method counting is shown in Fig. 9B. The curvature of the nematode at each body point (vertical axis) is a function of time (horizontal axis), with head curvature at the top and tail curvature at the bottom. Dark blue shows the curvature clockwise from the head and dark red shows the curvature counter-clockwise from the head. In order to make a better comparison between the proposed algorithm and CeleST method, the maximum distance between the peak point of nematode in each frame and the line between pharynx and tail is shown in Fig. 9C. The blue part represents the maximum distance clockwise from the head and the red part represents the maximum distance counter-clockwise from the head. Figure 9A shows the locomotion state of nematodes in regions I to III in Fig. 9B and C respectively. As can be seen from Fig. 9A (a), (b) and (c), the maximum distance of the nematode peak point is always on the same side, and the body bending count cannot move forward. However, the fringe number in region I in Fig. 9B is increased and the body bend count can be advanced. As can be seen from Fig. 9A (d), (e) and (f), the direction of the maximum distance between the nematode peak point and the line between the pharynx and the tail changed, and the results of the proposed method are consistent with those of the CeleST method for body bending counting. Counting results are shown in region II of Fig. 9B and C. As can be seen from Fig. 9A (g), (h) and (i), the nematode is in a deep bending state, where the curvature and maximum distance are shown in region III in Fig. 9B and C.

**Fig. 10:**
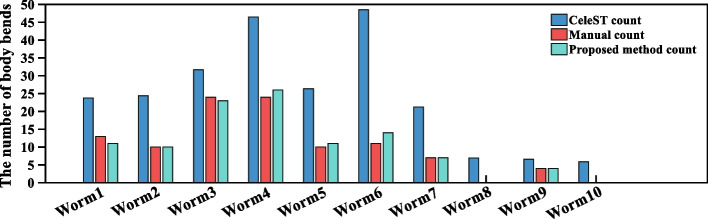
The results of the number of body bends are counted by CeleST method, manual and the proposed method respectively. The horizontal axis represents 10 worms. The vertical axis shows the number of body bends

Furthermore, in order to more accurately compare the effectiveness of the proposed method and CeleST method in calculating the number of body bends of nematodes, we conduct experiments on the demonstration dataset provided by CeleST method [18]. Nematodes in the dataset are swimming [18], and 10 nematodes are selected for the experiment. In order to compare the counting results of the two methods, we perform manual counting according to the criteria described in [22]. The results of the number of worm body bends calculated in three ways are shown in Fig. 10. As can be seen from Fig. 10, the number of body bends calculated by CeleST method is generally higher than that calculated by manual counting, and the counting results of the proposed algorithm are closer to manual counting. Experimental results show that the proposed algorithm is effective and robust.

### Applications for the proposed algorithm

The algorithm can automatically recognize and analyze the bending behavior of *C. elegans*. It can be used to evaluate the locomotion ability of *C. elegans* in toxicology, aging, drug screening, etc. Before using the tool, researchers could use filming equipment to capture video of individual *C. elegans*. Subsequently, the algorithm can be used to automatically recognize the body bending behavior of *C. elegans* and perform counting analysis. The algorithm can be used for counting under different illumination conditions. Of course, the better the illumination conditions, the higher the accuracy. Before feature point extraction, binary images need to be obtained. Generally speaking, the higher the resolution of the image, the better the segmentation effect. The running steps of the algorithm presented in this paper can be obtained from the Additional file 2.

However, this algorithm also has some limitations. For example, it may be impossible to accurately count some coiled behaviors of *C. elegans*. In addition, this algorithm can only be used to recognize and analyze the bending behavior of individual *C. elegans*. In the future, we will automatically recognize and analyze the bending behavior of multiple *C. elegans* in a video.

### Potential applications of the proposed algorithm

The proposed algorithm can be used not only to count the number of body bends but also to calculate the amplitude of worms. Worm amplitude is defined as the maximum amplitude found along the worm body as described in [19, 34]. In reference [19], the worm amplitude is quantified by fitting the entire body of the worm into a rectangular boundary box, and the width of the optimal boundary box is the amplitude. In reference [34], the skeleton of worm is rotated to the horizontal axis in the direction of an equivalent ellipse, with the origin being the centroid of the skeleton. The maximum y coordinate minus the minimum y coordinate is the maximum amplitude. In this paper, the maximum amplitude is obtained by calculating the angle between the peak point and two adjacent inflection points. The included angle *α*_*i*_ is shown in Fig. 3h. The smaller the angle *α*_*i*_, the greater the amplitude. Therefore, the smallest angle *α*_*i*_ is the maximum amplitude.

## Conclusion

The number of body bends is a key locomotion behavior indicator in assessing the locomotion capacity of nematodes. In order to reduce the manpower and time consumption in counting the number of nematode body bends and achieve high throughput and high accuracy counts, an algorithm is proposed for automatic counting and analysis of body bending behavior. The accuracy of the proposed algorithm is verified by manual counting. The effectiveness and robustness of the proposed method are verified by comparison with related methods. Different strains of nematodes are selected to analyze the differences in body bending behavior, which confirmed that nematodes show age-related decline in vitality [40–42]. Through high-throughput processing and analysis, the proposed method facilitates the study and characterization of nematodes’ locomotion behavior. Although we only select nematodes in foraging, escape, and swimming states for experiments to prove the robustness of the proposed algorithm, it is also applicable to nematodes in other states of locomotion. The proposed algorithm will provide convenience in the fields of drug activity screening, anti-aging research, and toxicological evaluation.

## Supplementary Information

**Additional file 1.** Specific experimental data of Caenorhabditis elegans behavioral phenotypes database and ‘escape response’ database.

**Additional file 2.** The specific operation steps of this method.

## Data Availability

The code is freely available at https://github.com/hthana/Body-Bend-Count.
