# Supplementary material for: Automated recognition and analysis of body bending behavior in C. elegans
Source: BMC Bioinformatics. 2023 Apr 28;24:175. doi: 10.1186/s12859-023-05307-y (PMC10148436; doi:10.1186/s12859-023-05307-y)

**###HeadTailReg-vgg19**

#Algorithm for head and tail localization is referenced from 'Mane M, Deshmukh A, Iliff A: Head and Tail Localization of C. elegans; 2020'. The relevant code can be obtained from 'https://github.com/mansimane/WormML'.

**# Runtime environment**

The operating system is Windows, the installed memory (RAM) is 8GB, the processor is Intel(R) Core(TM) i7-6700, and the CPU frequency is 3.40 GHz. The programming language is Python, the deep learning framework is tensorflow1.15.0, the development environment is PyCharm, and the development language version is Python3.6. Other Python configuration environments can be obtained from the file 'requirements.txt'.

**# Steps**

1. Run CreateDatasetV2.py - This file reads images and labels, splits images into train and validation data, detects worm bounding boxes and dumps 150x150 worm images. After the operation is completed, generate a training and validation dataset as well as normalized labels, as shown in the following figure.


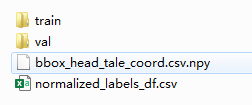


Normalized head and tail labels:


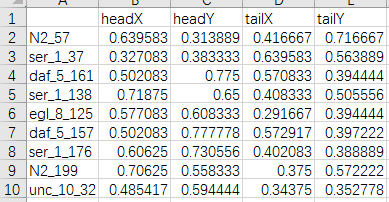


2. Run dsnt/wormml_dsnt.py - This file trains the model and dumps the predictions as well as tensorboard summaries in experiment folder. The final head and tail recognition results are shown in the following figure:


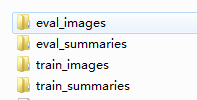


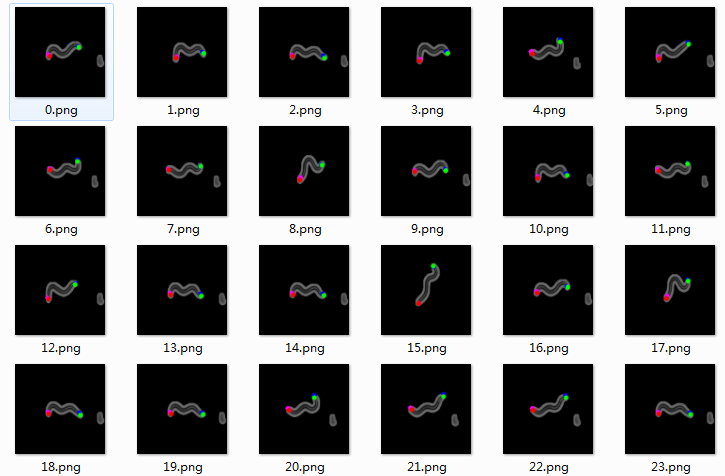


3. Place the first frame image of the experimental video in the validation dataset for head and tail coordinate prediction.

**###feature points extraction**

**# Runtime environment**

The operating system is Windows, the installed memory (RAM) is 8GB, the processor is Intel(R) Core(TM) i7-6700, and the CPU frequency is 3.40 GHz. The programming language is C++, the development environment is Visual Studio 2013.

**# Steps**

1. Double click to open feature points extraction.sln.


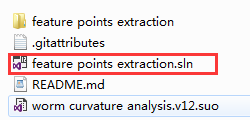


2. Click the button in the red box below to run the program.


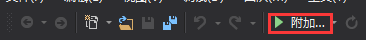


3. Obtain the coordinates of the pharynx, inflection point, and peak point. These results are shown in the three files in the figure below.


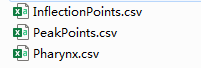


**###Count**

**# Runtime environment**

The operating system is Windows, the installed memory (RAM) is 8GB, the processor is Intel(R) Core(TM) i7-6700, and the CPU frequency is 3.40 GHz. The programming language is Matlab, the development environment is Matlab R2016a.

**# Steps**

1. Place a total of 4 ‘.csv’ files, including the head and tail coordinates calculated by the first algorithm and the feature point coordinates calculated by the second algorithm, in the Count folder.


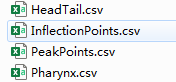


2. Open the program using Matlab R2016a, run ‘bendCount.m’, and obtain the experimental results.


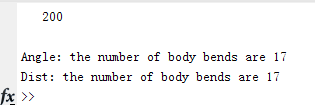

Supplement: Supplementary file 2 — Additional file 2. The specific operation steps of this method. [file 12859_2023_5307_MOESM2_ESM.docx]
